# Multimodal Nonlinear Microscopy for Therapy Monitoring of Cold Atmospheric Plasma Treatment

**DOI:** 10.3390/mi10090564

**Published:** 2019-08-26

**Authors:** Tobias Meyer, Hyeonsoo Bae, Sybille Hasse, Jörn Winter, Thomas von Woedtke, Michael Schmitt, Klaus-Dieter Weltmann, Juergen Popp

**Affiliations:** 1 Leibniz-Institute of Photonic Technology Jena, a member of the Leibniz Research Alliance Leibniz Health Technology, Albert-Einstein-Str. 9, 07745 Jena, Germany; 2 Abbe Center of Photonics, Friedrich-Schiller-University, Helmholtzweg 4, 07743 Jena, Germany; 3 Leibniz Institute for Plasma Science and Technology (INP), a member of the Leibniz Research Alliance Leibniz Health Technology, Felix-Hausdorff-Straße 2, 17489 Greifswald, Germany; 4 Department of Hygiene and Environmental Medicine, Medical Faculty, Greifswald University, Fleischmannstraße 8, 17475 Greifswald, Germany

**Keywords:** plasma medicine, cold atmospheric plasma, multimodal nonlinear imaging, two-photon fluorescence lifetime imaging, coherent Raman imaging

## Abstract

Here we report on a non-linear spectroscopic method for visualization of cold atmospheric plasma (CAP)-induced changes in tissue for reaching a new quality level of CAP application in medicine via online monitoring of wound or cancer treatment. A combination of coherent anti-Stokes Raman scattering (CARS), two-photon fluorescence lifetime imaging (2P-FLIM) and second harmonic generation (SHG) microscopy has been used for non-invasive and label-free detection of CAP-induced changes on human skin and mucosa samples. By correlation with histochemical staining, the observed local increase in fluorescence could be assigned to melanin. CARS and SHG prove the integrity of the tissue structure, visualize tissue morphology and composition. The influence of plasma effects by variation of plasma parameters e.g., duration of treatment, gas composition and plasma source has been evaluated. Overall quantitative spectroscopic markers could be identified for a direct monitoring of CAP-treated tissue areas, which is very important for translating CAPs into clinical routine.

## 1. Introduction

Defective wound healing affects millions of people in the western world. In particular due to the increasing abundance of multidrug-resistant bacteria, it poses a major public health challenge in developed countries, especially against the backdrop of an ageing population. There is therefore a great need for alternative therapies, especially for the treatment of chronic wounds. Recently, the direct application of cold atmospheric plasma (CAP) to or within the human body for therapeutic purposes has been shown to be very effective in the treatment of chronic and inflamed wounds, including wounds infected with multi-resistant microbes. By definition, physical plasma is an excited and conductive gas containing both differently charged and uncharged atoms and molecules, emitting electromagnetic radiation such as ultraviolet (UV), visible (VIS) and infrared (IR) radiation and other electromagnetic fields. CAP for medical applications is usually produced by applying an electric field to a neutral gas or gas mixture [1,2,3]. In addition to its antimicrobial activity, exposure of mammalian cells to CAP can either stimulate tissue regeneration and angiogenesis or inhibit cell function in cancer cells. Consequently, most research activities and first medical applications of physical plasma relate to wound healing and cancer treatment. First CAP sources got the European Conformity (CE) certification and comply with medical device regulations (Class IIa), mainly for the treatment of chronic wounds and infectious skin diseases [4,5,6].

One of the real limitations in controlling and optimizing medical CAP applications is the lack of appropriate methods for monitoring plasma effects on living tissue. The plasma devices currently available are based on two basic concepts: dielectric barrier discharges (DBD) and plasma jets [1,2,7,8,9]. While DBD plasma devices are suitable for generating plasma over larger areas and normally cover the treated area, a plasma jet device is moved over the surface to treat the whole area. Both devices lack direct plasma impact monitoring to ensure complete treatment of the entire wound or skin surface. This is particularly true for jet plasma devices, where the point-like plasma flow must be moved evenly and completely over the surface under treatment. In addition, endoscopic CAP devices are under development whose application also requires effective and meaningful therapy monitoring [10,11]. In addition to this basic therapy monitoring, i.e., the highlighting of tissue areas which have been exposed to plasma, there is also a lack of real-time visualization of therapeutic effects for the direct control of plasma treatment processes and their therapeutic consequences. Therefore, the successful introduction of CAP devices into clinical applications requires the realization of on-site real-time monitoring techniques of plasma effects. Currently, first attempts are being made to monitor CAP-based wound treatments with hyperspectral imaging techniques [12,13,14]. This enables the real-time visualization of physiological therapy effects, especially hemodynamic parameters resulting from complex plasma–tissue interactions. However, a specific non-invasive monitoring of interactions of plasma with defined tissue structures or components at the molecular level is still missing.

In recent years, a broad portfolio of spectroscopic methods has been investigated that allow a qualitative and quantitative evaluation of biochemical information both ex vivo and in vivo without the additional use of exogenous contrast agents. Most promising are combinations of several spectroscopic contrast mechanisms into a multimodal approach. In this context, it has been shown that multimodal nonlinear imaging, using different methods such as coherent anti-Stokes Raman scattering (CARS), two-photon excited autofluorescence (TPEF), two-photon excited fluorescence lifetime imaging (2P-FLIM) and second harmonic generation (SHG), is a powerful tool for the label-free characterization of the molecular composition of cells and tissues, enabling the visualization of the distribution of molecular markers with subcellular spatial resolution and the correlation of their function in tissue [15,16,17,18,19]. Using machine learning image processing algorithms, the nonlinear image data can be translated into biomedical information [20,21,22,23,24].

The effects of CAP on cells and tissues lead to redox-based changes in lipid and protein structures and to stimulation of redox-controlled and antioxidative cell pathways. The biological activity of CAP is mainly based on reactive oxygen and nitrogen species (ROS, RNS), which act together with other plasma components such as electromagnetic fields, UV and IR radiation [25,26,27,28,29]. A correlation between redox-based modifications of cell and tissue structures and compounds such as glutathione, cysteine residues and cell membrane lipids from plasma treatment was found [30,31,32]. With regard to in vivo wound healing processes, more detailed insights into redox-controlled molecular pathways triggered by plasma treatment have recently been reported [33,34,35]. This also applies to the plasma-based inactivation of cancer cells [36,37,38]. Important molecular targets for plasma-based biological effects are the Nrf2 signaling pathway, the cellular tumor antigen p53 or the cell membrane-bound calreticulin, the activation or suppression of which leads to several consecutive molecular reaction mechanisms. In order to monitor the effects of CAP treatment, first the distribution of abundant macromolecules needs to be studied to investigate the tissue morphology, structural changes and non-invasiveness of the treatment. These targets include collagen, cholesterol, lipids, keratins, elastic fibers and DNA, which can be investigated by CARS (lipids, DNA, keratin) SHG (collagen, cholesterol) and TPEF (elastic fibers, keratin). Structures and molecules of interest which are expected to be directly affected by CAP treatment include reactive oxygen species (ROS), flavin adenine dinucleotide (FAD) and flavins in general, reduced nicotinamide adenine dinucleotide (NAD(P)H) and melanin. Most of these molecules and structures are fluorescing. Hence, in the following contribution, multimodal nonlinear imaging combining CARS, SHG and 2P-FLIM has been employed to investigate the effects of CAP treatment on tissue for the first time.

## 2. Materials and Methods

### 2.1. Specimen

Human skin samples were obtained from routine surgery in the department of dermatology at the University Medicine Rostock, Rostock, Germany. Patients signed written consent to participate in the laboratory study and permitted the non-invasive collection of tissue samples for additional histological examinations. The local ethical committee approved. For the experiments, 3 mm punch biopsies from the healthy rim of an excised lesion of skin and mucosa were used. First, the tissue sample was frozen onto a sample holder of a cryostat, then 6 µm sections were cut and immediately transferred to a quartz slide for further analyses.

### 2.2. Plasma Treatment

**Figure 1:**
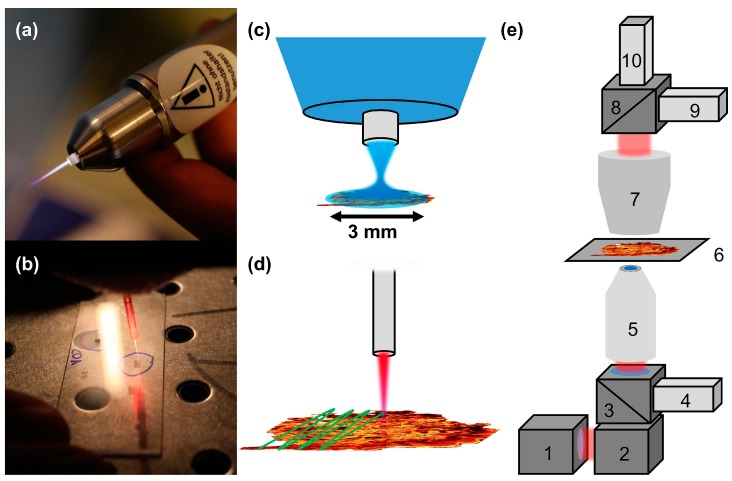
Photographs of kINPen-device^®^ (**a**) and endoscopic plasma device (**b**) during operation; (**c**) Sketch of treatment of a thin tissue section using the kINPen-device^®^ as shown in (**a**). The plasma covers the whole area of the tissue sections under investigation, approximately 3 × 3 mm² in size. (**d**) The plasma jet of the endoscopic plasma device shown in panel (**b**) is moved across the area of the specimen for treatment of the full section. (**e**) Scheme of the setup used for multimodal nonlinear microscopy combining coherent anti-Stokes Raman scattering (CARS), second harmonic generation (SHG) and two-photon fluorescence lifetime imaging (2P-FLIM). The picosecond (ps) pulse trains of the Ti: sapphire laser/optical parametric oscillator (OPO) system (1) is coupled into the laser scanning microscope (2). The laser light is focused onto the sample by a microscope objective (5) for image acquisition by scanning the specimen (6). Two-photon excited autofluorescence (TPEF) signals are collected by the objective (5) and reflected to the 2P-FLIM detector (4) by a 600 nm short pass dichroic mirror (3). The TP-FLIM signal is filtered from residual laser light by a 650 nm short pass filter and a 458/64 nm bandpass filter (both Semrock, Rochester, MN, USA). The CARS and SHG signals from the sample are collected in a forward direction by a condenser (7), split by a 514 nm long pass dichroic mirror and detected by photomultiplier tube (PMT) modules (9 SHG, 10 CARS) after spectral filtering as described in Section 2.3.

The experimental procedure of plasma treatment is schematically depicted in Figure 1a–d. Thin tissue sections from human skin and mucosa were treated with plasma for a specific time using two different plasma sources. In Figure 1a,c the certified medical device kINPen MED^®^ (neoplas tools GmbH, Greifswald, Germany) is shown [39]. Tissue samples were directly exposed to the plasma effluent at a distance of about 8 mm from the capillary outlet covering the whole area of the tissue section of 3 × 3 mm². The gas temperature at working distance was 35–39 °C. The second CAP device used is shown in Figure 1b,d. This endoscopic plasma source has been described in detail elsewhere [10,11]. In order to treat the complete tissue section with the endoscopic plasma device, the plasma jet was moved across the whole sample (see Figure 1d). The treatment time is the overall time including movement of the plasma jet across the specimen as depicted in Figure 1d. For the plasma treatment, several parameters were varied: the plasma source, the treatment time and the gas composition. The kINPen MED^®^ has been applied using an Argon gas flow of 4200 standard cubic centimeters per minute (sccm). In an additional setting, 10.5 sccm oxygen was admixed to the basic Ar feed gas (0.25% admixture). In the endoscopic plasma source, Neon feed gas with a gas flow rate of 300 sccm was applied. Similar to the kINPen MED^®^ experiments, oxygen with a gas flow rate of 3 sccm was admixed to the Ne feed gas in the endoscopic plasma source (1% admixture). The gas flows were controlled by mass flow controllers MFC 1179 (MKS Instruments, Andover, MA, USA). Treatment times of 10 s up to 70 s were used.

Differences and changes in temperature can be ruled out. All tissue samples were cut at 6 µm thin slices and placed immediately on quartz slides at room temperature. At the time of plasma treatment, the tissue was completely adapted to the ambient temperature. The tissue sections were defrosted throughout. In order to minimize the influence of time, the images of untreated and treated samples were obtained in a short sequence. Therefore, the control image was taken directly before and the final image directly after the plasma treatment. The total sequence time (control image, plasma treatment, final image) took only a couple of minutes. No severe changes were observed when an untreated sample was probed two times within this measurement period. Thus, a sequence time influence on the observed changes is very unlikely. We also investigated two arbitrarily chosen samples one day after CAP treatment and found that the CAP treatment induced changes in autofluorescence were within the accuracy of the experiment the same as the day before. Hence, CAP treatment induced changes in autofluorescence are persistent.

### 2.3. Multimodal Nonlinear Imaging Experiments

In order to search for novel markers for monitoring CAP treatment, tissue sections were investigated by multimodal nonlinear microscopy using CARS, SHG and 2P-FLIM preceding and subsequent to the CAP treatment. The nonlinear imaging data measured from the tissue samples before CAP treatment served as negative control. The setup for performing multimodal nonlinear microscopy is schematically depicted in Figure 1e. The setup for simultaneous acquisition of CARS, TPEF and SHG signals has been described in detail previously [40]. Briefly, the laser source (depicted in Figure 1e number 1) consists of a picosecond (ps) Ti: sapphire laser (Mira HP, Coherent, Santa Clara, CA, USA). The laser output is split into two parts. The first part is used to pump an optical parametric oscillator (OPO, APE, Berlin, Germany) for generating the tunable pump laser for CARS microscopy by parametric conversion in a periodically poled lithium niobate crystal followed by SHG of the signal wavelength. The OPO output is recombined with the second fraction of the Ti:sapphire laser output and spatially and temporally overlapped for CARS imaging. Here, the Ti: sapphire laser is used as Stokes laser. For CARS imaging of the symmetric aliphatic CH-stretch vibration of methylene groups CH_2_ at 2850 cm^−1^ the SHG of the OPO signal and the Ti: sapphire laser are used and the lasers are tuned to 671.1 and 832.2 nm, respectively.

The laser beams are coupled into a laser scanning microscope (see Figure 1e number 2, LSM510, Zeiss, Jena, Germany). The laser passes a long pass dichroic mirror (see Figure 1e number 3, long pass 600 nm, Zeiss, Germany) before being focused onto the specimen by a 20× microscope objective (see Figure 1e number 5, Plan-Apochromat, NA 0.8, Zeiss, Germany). The fluorescence signal is collected by the microscope objective and filtered from the laser light by the dichroic mirror (see Figure 1e number 3) and two filters (short pass 650 nm, bandpass 458/64 nm, Semrock, USA) before detection by the FLIM module (see Figure 1e number 4, Becker & Hickl, Berlin, Germany). Alternatively, a non-descanned photomultiplier tube (PMT) can be used for detection of the TPEF signal [41]. The 2P-FLIM system consists of a hybrid GaAsP detector (HPM-100-40, Becker & Hickl, Germany) and a workstation containing a time correlated single photon counting (TCSPC) module (SPC-150, Becker & Hickl, Germany). Typical 2P-FLIM imaging parameters are 512 × 512 pixels, 1024 time channels, time range 12.51 ns, acquisition time 70–200 s. The laser powers at the samples are adjusted to reduce the count rate of the FLIM detector below 1 MHz using approximately 10 mW of pump and 25–50 mW of Stokes laser power at the sample (see Figure 1e number 6). The 2P-FLIM module enables precise quantification of the TPEF signals by single photon counting. CARS and SHG signals are predominantly emitted in a forward direction. Hence, the CARS and SHG signals are collected by a condenser (see Figure 1e number 7, NA 0.8, Zeiss, Germany), the signals are split by a dichroic beam splitter (see Figure 1e number 8, HFT 514, Zeiss, Germany), filtered (CARS: Short pass 650 nm, SHG: Bandpass 415 nm) and detected by photomultiplier tubes (PMT). SHG and CARS signals are detected by PMT detectors (see Figure 1e; SHG-detector 9, CARS detector 10).

### 2.4. Staining Histopathology

The demonstration of melanin in the tissue sections after CAP treatment and multimodal nonlinear imaging was performed by Fontana–Masson staining (AgNO_3_).

## 3. Results

**Figure 2:**
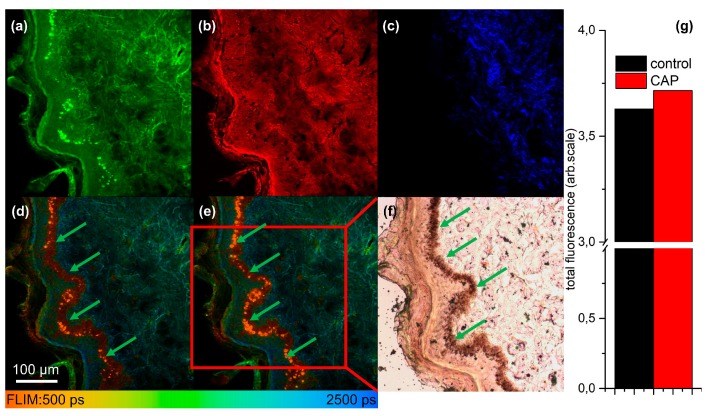
Multimodal nonlinear imaging of a human skin tissue sample before (panels a–d) and after cold atmospheric plasma (CAP) treatment for 20 s using the kINPen-device with an Ar–oxygen mixture (panel e). (**a**) TPEF image, excitation at 672.5 nm and 832.2 nm due to simultaneous CARS imaging using these wavelengths as pump and Stokes, emission 426–490 nm; (**b**) CARS image at 2850 cm^−1^; (**c**) SHG image at 415 nm; (**d**) 2P-FLIM image for the same parameters as in (**a**) before CAP treatment, the green arrows mark melanocytes within the stratum basale; (**e**) 2P-FLIM image for the same parameters as in (**a**) after 20 s of CAP treatment using the kINPen MED^®^-device with an Ar–oxygen mixture revealing an overall increase in fluorescence, particularly in the melanocytes inside the stratum basale, indicated by green arrows; (**f**) brightfield microscopic image of AgNO_3_ staining for melanin proving localization of the CAP-induced increase of autofluorescence within the melanocytes inside the stratum basale, marked by green arrows. (**g**) total fluorescence signal before and after CAP treatment. The fluorescence intensity increases in this case by 2%, typically the increase is larger, see Figure 4a.

**Figure 3:**
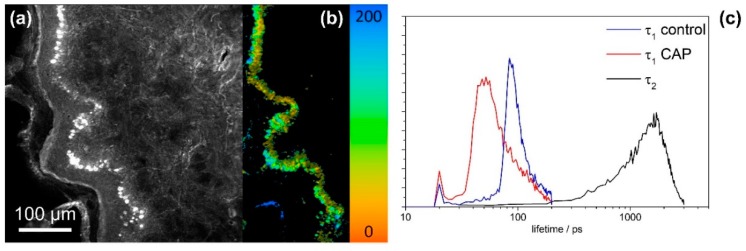
(**a**) TPEF image of the human skin tissue section treated with the kINPen MED^®^ plasma device for 10 s with an Ar–oxygen working gas. Excitation at 670 nm, emission 458/64 nm. (**b**) 2P-FLIM image of lifetime component t_1_ fitting the data of (a) with a bi-exponential decay function using a threshold of 20 and bin 1 in order to fit the brightest pixels within the melanocytes inside the stratum basale only. (**c**) Histograms of the lifetime t_1_ before and after CAP treatment and of t_2_ after CAP treatment.

In order to search for biomarkers for monitoring the CAP treatment, multimodal nonlinear imaging combining CARS, SHG and 2P-FLIM was applied to investigate thin tissue sections of skin and mucosa. The sections were analyzed before and after CAP treatment to search for spectroscopic changes related to the CAP treatment. Results from imaging CAP-induced spectral changes are displayed in Figure 2 for a human skin tissue sample treated with the kINPen MED^®^ device by an Ar–oxygen gas mixture for 20 s. In Figure 2a–d TPEF (excitation at 672.5 nm and 832.2 nm, emission 426–490 nm), CARS, SHG and 2P-FLIM images of the tissue specimen before CAP treatment are compared to changes in the fluorescence lifetime and intensity after treatment in panel e. It is evident that the autofluorescence of the specimen increases after treatment (see Figure 2g). The increase of autofluorescence is mainly localized in the proliferating and metabolically active basal cell layer, i.e., the melanocytes inside the stratum basale. These cells are also rich in melanin (see green arrows in Figure 2) as evident by the comparison with AgNO_3_ staining for melanin proving localization of the CAP-induced increase of autofluorescence within the melanin layer (see Figure 2f). Similar results have been obtained for the endoscopic CAP device (see Figure S2a,f). In order to prove that the observed strong fluorescence within the epithelium is due to melanin, melanin was selectively excited using the pump laser at 670 nm only, which best fits the melanin two-photon absorption. The melanin two-photon absorption is spectrally broad but decreases from 300 to 700 nm. The emission peaks from 450 to 600 nm, such that the filter used fits the melanin emission range [42]. The fluorescence lifetime of melanin has been reported to be in the range of 100–150 ps [43]. In Figure 3a, the TPEF image of the human tissue section excited at 670 nm is displayed. The 2P-FLIM-data was fitted with a bi-exponential decay function using a threshold of 20 and bin 1 in order to fit the brightest pixels within the melanocytes inside the stratum basale only. The 2P-FLIM image of the short lifetime component is displayed in Figure 3b. Excitation at 670 nm results in bright emission from the basal cell layer characterized by a first lifetime component of 50 ps, much lower than the lifetime of free NAD(P)H of 400 ps [44]. The histograms of the lifetime t_1_ and t_2_ are shown in Figure 3c.

For the control, the first lifetime t_1_ is about 100 ps, while it is reduced to about 50 ps for the CAP treatment, fitting the fluorescence lifetime of melanin well. The second lifetime t_2_ is about 1800 ps for the CAP treated specimen, approximately fitting to protein bound NAD(P)H. These findings suggest that CAP treatment induces a prominent increase specifically of melanin fluorescence and a significant shift of the melanin lifetime (see Figure 3c). This is possibly due to the generation of reactive species, which provide additional deactivation for excited melanin, which results in a reduction of lifetime.

**Figure 4:**
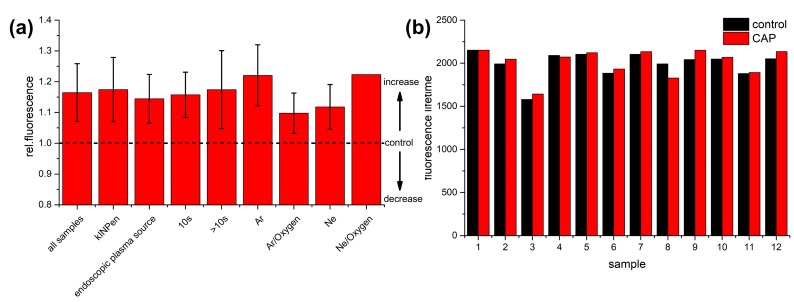
Results from two-photon time correlated single photon counting (TCSPC) fluorescence measurements (**a**) The relative change of the total fluorescence (two-photon excitation at 672.5 nm and 832.2 nm, corresponding to single photon excitation at 336 and 416 nm, fluorescence emission at 426–490 nm) after CAP treatment is plotted for all samples normalized to the total fluorescence of the control (=1.0), i.e., all measurements and experimental conditions (treatment time, plasma source, gas composition, specimen), the two plasma devices kINPen MED^®^ (using Ar and an Ar–oxygen gas mixture for operation and treatment times from 10–70 s) and the endoscopic plasma device (using Ne and a Ne–oxygen gas mixture for operation and treatment times of 10 and 60 s), treatment times of 10 s (in combination with different CAP devices and gas mixtures), longer treatment times (20 s, 60 s and 70 s) and different gas mixtures. Here, Ar and Ar–oxygen gas mixtures were used with the kINPen-device only, Ne and Ne–oxygen gas mixtures were used with the endoscopic plasma device only. (**b**) Change of the median fluorescence lifetime for the parameters of (**a**) for all samples under investigation and all 12 experimental conditions using a monoexponential decay function and plotting the median lifetime of the whole FLIM image. The experimental conditions are in detail: 1,2: Skin, 10 s kINPen, Ar gas; 3: Mucosa lesion, 10 s kINPen, Ar gas; 4: Mucosa, 10 s kINPen, Ar gas; 5,6: Skin, 10 s kINPen, Ar–oxygen gas mixture; 7: Skin, 20 s kINPen, Ar–oxygen gas mixture; 8: Skin, 70 s kINPen, Ar gas; 9: Skin, 10 s endoscopic plasma source, Ne gas; 10,11: Skin, 1 min endoscopic plasma source, Ne gas; 12: Skin, 1 min endoscopic plasma source, Ne–oxygen gas. In eight cases a lifetime increase was observed, while in four cases the fluorescence lifetime was reduced.

Apart from melanin, further endogenous fluorophores are observed for the applied excitation and emission window, in particular NAD(P)H, collagen and elastin [45]. In the following, different CAP devices were used and treatment parameters varied, and the sample fluorescence was analyzed in order to investigate, whether the increase in fluorescence is potentially a general readout parameter for CAP treatment monitoring. For these experiments, two CAP devices, the kINPen MED^®^ and an endoscopic plasma source were employed. The kINPen MED^®^ was used with Ar and Ar with 0.25% oxygen as feed gas applied to mucosa and skin specimens for treatment times from 10–70 s. The endoscopic plasma device was used with Ne and Ne with 1% oxygen admixture as working gas and applied for 10 or 60 s to human skin specimens. First, for all these experimental conditions an overall increase in autofluorescence has been observed (see Figure 4a, first column). The total fluorescence increase is observed for both devices (see Figure 4a, second and third column labeled kINPen and endoscopic plasma source).

When comparing the plasma devices, the fluorescence increase was slightly larger for the kINPen MED^®^ plasma device in comparison to the plasma endoscope. However, since both devices use different gases for operation, this effect may be solely due to the different working gas, because the fluorescence increase was larger for Ar in comparison to Ne gas (see Figure 4a, columns labeled Ar and Ne). Additionally, the gas jet is much smaller for the endoscopic device, such that the endoscopic device is moved across the sample for treatment, while the plasma jet of the kINPen MED^®^ plasma device covers the whole specimen. Hence, the effective treatment time is lower for the endoscopic device, which may account for the reduced effect as well. The fluorescence increase is directly correlated with the treatment time as is evident when comparing experiments with treatment times of 10 s with those of longer treatment (see Figure 4a, columns labeled 10 s and >10 s): The fluorescence increase is larger if the CAP treatment is performed for a longer time. For the kINPen MED^®^ plasma device, the increase in fluorescence was larger for pure Ar gas while the addition of oxygen resulted in a lower increase of autofluorescence (see Figure 4a, columns labeled Ar and Ar/Oxygen). This is most likely due to the fact, that oxygen improves the recombination of charges in the plasma. For the plasma endoscope the reverse effect was observed. The fluorescence increase was larger for a mixture of Ne and oxygen compared to pure Ne (see Figure 4a, columns labeled Ne and Ne/Oxygen). However, this finding is based on a single experiment for the Ne/O_2_ mixture. Therefore, the result can be due to differences in the sample response.

In order to investigate sample integrity and search for macroscopic morphologic changes, SHG and CARS microscopy were performed in parallel to 2P-FLIM. Significant morphologic changes were not observed comparing CARS and SHG images acquired before and after CAP treatment for both CAP devices (see supporting information Figure S1 and S2). Hence, CAP treatment is not altering the sample morphology.

While a significant increase in fluorescence within the epithelium was assigned to melanin and a reduction in melanin lifetime was observed, all experimental conditions were analyzed in order to search for characteristic lifetime changes in general. When investigating the whole treated area, the mean fluorescence lifetime for two-photon excitation using 670 nm and 830 nm, which corresponds to one-photon excitation at 335 and 415 nm, no clear change in median fluorescence lifetime was observed within the observed emission window from 426–490 nm (see Figure 4b). Here, a monoexponential decay function was fitted to the data and the mean fluorescence lifetime was calculated in each pixel. The median fluorescence lifetime of the whole image was calculated and is plotted for different treatment conditions (see Figure 4b: 1,2: Skin, 10 s kINPen, Ar gas; 3: Mucosa lesion, 10 s kINPen, Ar gas; 4: Mucosa, 10 s kINPen, Ar gas; 5,6: Skin, 10 s kINPen, Ar–oxygen gas mixture; 7: Skin, 20 s kINPen, Ar–oxygen gas mixture; 8: Skin, 70 s kINPen, Ar gas; 9: Skin, 10 s endoscopic plasma source, Ne gas; 10,11: Skin, 1 min endoscopic plasma source, Ne gas; 12: Skin, 1 min endoscopic plasma source, Ne–oxygen gas). For 8 of 12 experimental conditions, a slight increase in lifetime was observed, while the lifetime was reduced in four cases. Hence, in contrast to a clear reduction of the lifetime of the melanin fluorescence due to CAP treatment, no specific lifetime changes were observed for the overall fluorescence of skin and mucosa samples. Apart from melanin, the total fluorescence also originates from NAD(P)H, collagen and elastin. Therefore, that the fluorescence lifetime is reduced due to CAP treatment is not a general trend for all fluorescing molecules.

## 4. Discussion

The key results from multimodal nonlinear imaging combining CARS, SHG and 2P-FLIM microscopy for CAP treatment monitoring are as follows. First, CAP treatment induces a significant increase of the overall autofluorescence signal, which can be attributed to fluorescence from melanin, NAD(P)H, FAD, elastin and collagen within the spectral emission window from 426–490 nm. In general, no significant change of fluorescence lifetime has been observed for the ensemble of endogenous fluorophores. Therefore, CAP-induced changes in fluorescence lifetime need to be specifically discussed and investigated for the individual fluorophores. However, within the accuracy of our experiments and the experimental conditions enabling excitation of melanin, NAD(P)H, FAD, elastin and collagen, we were able to assign the increase of fluorescence within the melanocytes inside the stratum basale to melanin. The melanin autofluorescence within the basal cell layer significantly increases due to CAP treatment and the lifetime of the melanin fluorescence is reduced. At the present state of knowledge, it can be speculated only about the chemical correlates of these effects on melanin fluorescence. Both changes in the melanin environment and the CAP-induced formation of reactive species, which provide additional non-radiating relaxation pathways of excited melanin, may be responsible for the observed reduction in fluorescence lifetime of melanin. The fact that the exact structure of melanin is not conclusively explained yet additionally complicates this. It is known that melanin is a copolymer consisting of indole subunits that could be target structures for redox-based changes which could finally result in changes of fluorescent properties of the molecule [46]. So far, the increase in autofluorescence within other anatomic structures than the stratum basale could not be precisely assigned to specific fluorescing molecular species. Here, longer integration times would be needed in order to collect ~10^5^ photons per pixel for individual decay traces enabling fitting of up to three decay components to assign the observed changes to individual fluorophores. However, such long integration times would have significantly enhanced the photon load of the specimen, which also results in an increase in autofluorescence [47]. The tissue morphology as monitored by CARS and SHG imaging is not affected by CAP treatment, which clearly proves the non-invasiveness of the method as it preserves the tissue morphology. Even though a clear correlation of the autofluorescence increase with the CAP treatment time has been observed, even a short treatment of 10 s is already sufficient for generating a clear increase in the autofluorescence signal. Hence, it can be concluded that CAP treatment is effective even for short application, which is highly promising for routine application in clinical practice.

While different gas mixtures can be used for plasma generation, i.e., Ne, Ar and mixtures with oxygen, the fluorescence increase was larger for Ar in comparison to a mixture of Ar and oxygen as observed for the kINPen MED^®^ plasma device. The effect was larger for Ar than for Ne, which may be also related to the different size of the plasma jet and the different plasma source. For the plasma endoscope the reverse effect was observed for Ne and a mixture of Ne with oxygen. However, this was the result from a single measurement only and needs to be confirmed on a larger set of experiments. The endoscope and handheld kINPen MED^®^ device generate similar results, however, the fluorescence increase was slightly larger for the kINPen MED^®^ plasma device in comparison to the plasma endoscope. Since many other factors may contribute to this finding, e.g., the plasma jet is much larger in the kINPen MED^®^ and therefore the effective treatment time of the tissue area is longer, the working gas is different for the endoscope (Ne) and kINPen MED^®^ (Ar), further investigations are needed to investigate the observed differences in detail.

The results presented in this paper are a first proof-of-concept to demonstrate the general possibility of visualizing CAP-induced tissue changes by label-free multimodal nonlinear microscopy. It is hypothesized that changes in fluorescence are results of redox-based modifications of molecular structures of specific molecules like melanin. On the other hand, it is well-known that CAP-supported wound healing as well as inactivation of cancer cells are mainly redox-controlled processes [33,34,35,36,37,38]. Consequently, the aim of further research is to identify tissue components and structures that are connected to specific biological responses like tissue regeneration or cancer cell inactivation that can be visualized by label-free spectroscopic methods to correlate their changes on a molecular level in tissue with subsequent biological effects. The results presented are a first step only to demonstrate the general feasibility of this concept. However, any correlation to CAP-induced wound healing processes or other specific biological effects of CAP are not possible to determine at the present state of research. Moreover, any transfer of results from investigations of tissue samples ex vivo to the situation in the living tissue in vivo needs much more research including clinical investigations.

## 5. Conclusions

It has been demonstrated that multimodal nonlinear microscopy combining SHG, CARS, TPEF and 2P-FLIM is a powerful diagnostic tool to monitor and investigate the mechanisms of CAP treatment on molecular level without using exogenous labels. By using specific excitation and emission wavelengths adapted to autofluorescing marker molecules, we believe that the signals which correlate with CAP treatment can be significantly increased and characteristic lifetime changes can be observed and correlated to specific molecular markers. 2P-FLIM is a highly valuable tool to investigate the generation of reactive oxygen species (ROS) induced by CAP-treatment, which modify the endogenous fluorescence, e.g., by activating emission-free de-excitation pathways. 

## Supplementary Materials

The following are available online at https://www.mdpi.com/2072-666X/10/9/564/s1, Figure S1: Multimodal nonlinear image of human skin tissue sample of TPEF (a), CARS (b) and SHG (c) before (upper row) and after CAP treatment (d–f) using the kINPen plasma device for 10 s with an Ar–oxygen working gas mixture. No morphologic changes were observed apart from an increase in autofluorescence. Figure S2: Multimodal nonlinear image of human skin tissue sample of 2P-FLIM (a), TPEF (b), CARS (c) and SHG (d) before (upper row) and after CAP treatment (lower row, same color code) using the endoscopic plasma device for 60 s with Ne working gas. Within the CARS and SHG channel, no morphologic changes have been observed apart from a significant increase in autofluorescence within the basal cell layer, which also leaks into the CARS image (550 nm).
