# Supplementary material for: Multimodal Nonlinear Microscopy for Therapy Monitoring of Cold Atmospheric Plasma Treatment
Source: Micromachines (Basel). 2019 Aug 26;10(9):564. doi: 10.3390/mi10090564 (PMC6780561; doi:10.3390/mi10090564)
Supplement: Supplementary file 1 [file micromachines-10-00564-s001.pdf]

micromachines

Supporting information

Multimodal Nonlinear Microscopy for Therapy Monitoring of Cold Atmospheric Plasma Treatment

Tobias Meyer, Hyeonsoo Bae, Sybille Hasse, Jörn Winter, Thomas von Woedtke, Michael Schmitt, Klaus-Dieter Weltmann and Juergen Popp

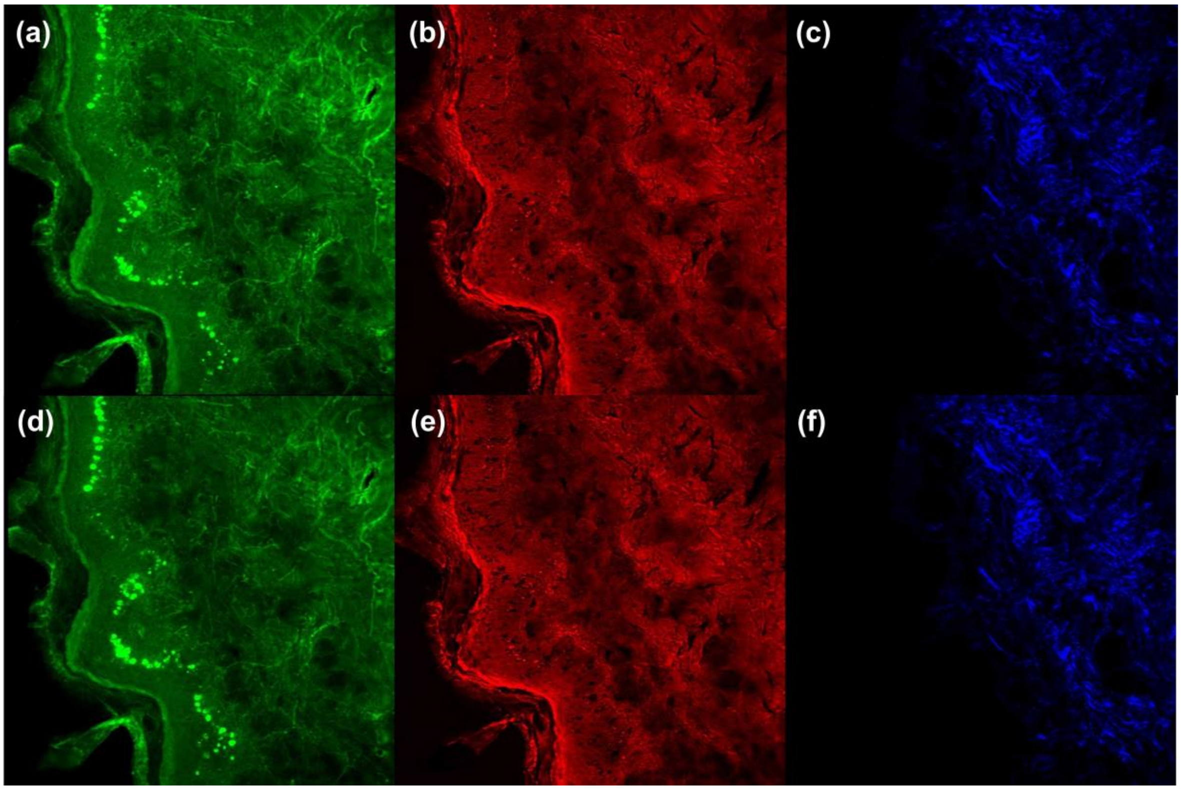

Figure S1. Multimodal nonlinear image of human skin tissue sample of TPEF (**a**), CARS (**b**) and SHG (**c**) before (upper row) and after CAP treatment (**d**–**f**) using the kINPen plasma device for 10 s with an Ar-oxygen working gas mixture. No morphologic changes have been observed apart from an increase in autofluorescence.

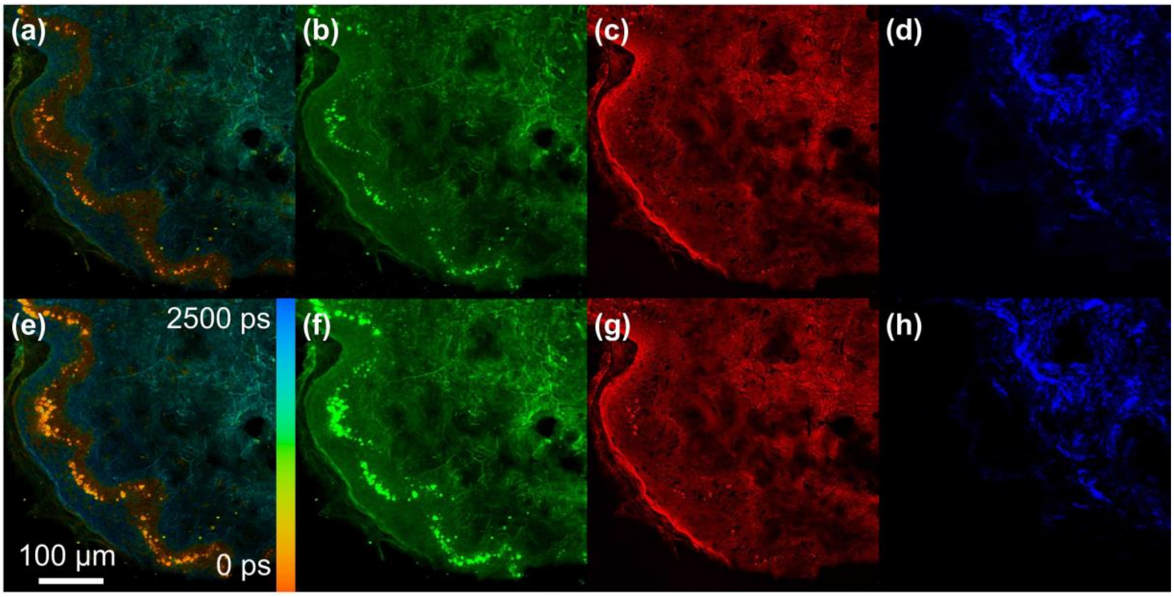

Figure S2. Multimodal nonlinear image of human skin tissue sample of 2P-FLIM (**a**), TPEF (**b**), CARS (**c**) and SHG (**d**) before (upper row) and after CAP treatment (lower row, same color code) using the endoscopic plasma device for 60 s with Ne working gas. Within the CARS and SHG channel, no morphologic changes have been observed apart from an significant increase in autofluorescence within the basal cell layer, which also leaks into the CARS image (550 nm).

© 2019 by the authors. Licensee MDPI, Basel, Switzerland. This article is an open access article distributed under the terms and conditions of the Creative Commons Attribution (CC BY) license (http://creativecommons.org/licenses/by/4.0/).
